# Time makes histone H3 modifications drift in mouse liver

**DOI:** 10.18632/aging.204107

**Published:** 2022-06-10

**Authors:** Roman Hillje, Lucilla Luzi, Stefano Amatori, Giuseppe Persico, Francesca Casciaro, Martina Rusin, Mirco Fanelli, Piergiuseppe Pelicci, Marco Giorgio

**Affiliations:** 1 Department of Experimental Oncology, IRCCS - European Institute of Oncology, Milano 20139, Italy; 2 Department of Biomolecular Sciences, Molecular Pathology Lab, University of Urbino 'Carlo Bo', Fano 61032, Italy; 3 Department of Oncology and Hemato-Oncology, University of Milan, Milano 20122, Italy; 4 Department of Biomedical Sciences, University of Padua, Padova 35131, Italy

**Keywords:** epigenetics, aging, histones, ChIP-seq, diet

## Abstract

To detect the epigenetic drift of time passing, we determined the genome-wide distributions of mono- and tri-methylated lysine 4 and acetylated and tri-methylated lysine 27 of histone H3 in the livers of healthy 3, 6 and 12 months old C57BL/6 mice. The comparison of different age profiles of histone H3 marks revealed global redistribution of histone H3 modifications with time, in particular in intergenic regions and near transcription start sites, as well as altered correlation between the profiles of different histone modifications. Moreover, feeding mice with caloric restriction diet, a treatment known to retard aging, reduced the extent of changes occurring during the first year of life in these genomic regions.

## INTRODUCTION

Aging is known to involve epigenetic histone modifications which are associated with transcriptional changes occurring throughout the entire lifespan of an individual.

More than a thousand post-translational histone modifications, also known as histone marks, have been identified, many of which are associated with different chromatin states [1]. Tri-methylated lysine 4 and lysine 27 of histone H3, i.e. H3K4me3 and H3K27me3, respectively, are two of the most investigated histone marks. High levels of H3K4me3 are found in chromatin domains at the promoters of actively transcribed genes in mouse [2] as well as human [3] and are associated with unmethylated CpG regions [4]. In contrast, H3K27me3, targeted by the Polycomb methyltransferase repressive complex [5], is found mainly on silenced loci in mouse [6] and human [7] and is considered a hallmark of a transcription-repressive chromatin state [8]. The acetylation of lysine 27 of histone H3 (H3K27ac) and the mono-methylation of lysine 4 of histone H3 (H3K4me1) are associated with active enhancers [9] and thereby also linked to transcription.

Several studies suggest that aging induces an overall drift of epigenetic signals in all organisms [10, 11]. Consistent with an increased fraction of heterochromatin observed in elderly individuals of different species [12], Wood and colleagues reported a reduction of histone marks associated with active chromatin, including tri-methylated lysine 4 of histone H3 (H3K4me3), and an increase of those histone marks linked to silencing of transcription, such as H3K27me3, in old *D. melanogaster* [13]. Moreover, *D. melanogaster* mutants defective for the H3K27 methylase, with reduced global H3K27me3 levels, resulted to be long-lived [14]. Likewise, *C. elegans* with increased activity of the H3K4 methylase ASH-2/trithorax complex 9 and high levels of H3K4me3 showed accelerated aging [15]. However, the overall amount of H3K27me3 in *C. elegans* was reported to decrease with age, and RNA interference of the H3K27me3 demethylase UTX-1 prolonged lifespan by suppressing the expression of genes of the insulin pathway [16]. In contrast to *D. melanogaster*, both higher levels of H3K4me3, resulting from inactivation of the major H3K4me3 methyltransferase complex, and lower levels of H3K4me3, upon suppression of demethylases, are associated with longevity in *C. elegans* [15, 16].

In mice, H3K27me3 was found to increase in muscle quiescent stem cells from aged individuals and, thus, is suggested to suppress functions related to stemness [17]. Furthermore, hematopoietic and muscle stem cells have been observed to accumulate H3K4me3 with age [18]. Particularly high levels of H3K27me3 were observed in the brain of mice with an accelerated aging phenotype [19]. Yet, H3K27me3 levels appear to decrease with age, while H3K4 tri-methylation to increase at specific regions, such as the Ink4a/Arf anti-proliferative locus in pancreatic islets [20]. Global histone acetylation was found regulated in aged mice brain as well [21]. In the mouse liver, nucleosome occupancy [22], the overall content of several histone marks [23] and chromatin remodeling has been suggested to occur throughout lifespan [24]. So far, one study reported progressive changes in genome-wide distribution of histone marks (H3K4me3 and H3K27ac) during mammalian aging [25].

In humans, H3K4me3 distribution in prefrontal neurons from 11 individuals is the only genome-wide histone mark study reported throughout aging up to now. From this analysis, H3K4me3 resulted to decrease in 600 loci in early life and to increase in other 100 loci in aged adults [26]. So far, no study discloses any drift of histone marks in mammals which is time-dependent or influenced by pro-longevity caloric restriction treatment.

In this study, we used chromatin immunoprecipitation sequencing technology to acquire 108 high-resolution profiles of H3K4me3, H3K4me1, H3K27me3 and H3K27ac from the livers of mice aged between 3 months and 12 months and fed 30% caloric restriction diet (CR) or standard diet (SD).

While C57BL/6 mice can become significantly older, 12 months was chosen as the age maximum in this study to focus on the changes in histone modifications which occur in the absence of age-related dysfunctions.

## RESULTS

### Global patterns of histone mark profiles identified age and diet groups

Livers were obtained from C57BL/6 female mice of 3, 6 and 12 months of age fed with standard diet (SD) and 12 months of age fed with 30% caloric restriction (CR). We immunoprecipitated and obtained histone profiles for H3K4me3, H3K27me3, H3K27ac and H3K4me1 for a total amount of 108 sequences. In particular, we produced 30 profiles for the group SD 3m (7 H3K4me3, 7 H3K27me3, 8 H3K27ac, 8 H3K4me1), 20 for SD 6m (3 H3K4me3, 6 H3K27me3, 6 H3K27ac, 5 H3K4me1), 29 for SD 12m (8 H3K4me3, 8 H3K27me3, 7 H3K27ac, 6 H3K4me1) and 29 for CR 12m (5 H3K4me3, 6 H3K27me3, 9 H3K27ac, 9 H3K4me1) as summarized in the Supplementary Table 1.

**Figure 1:**
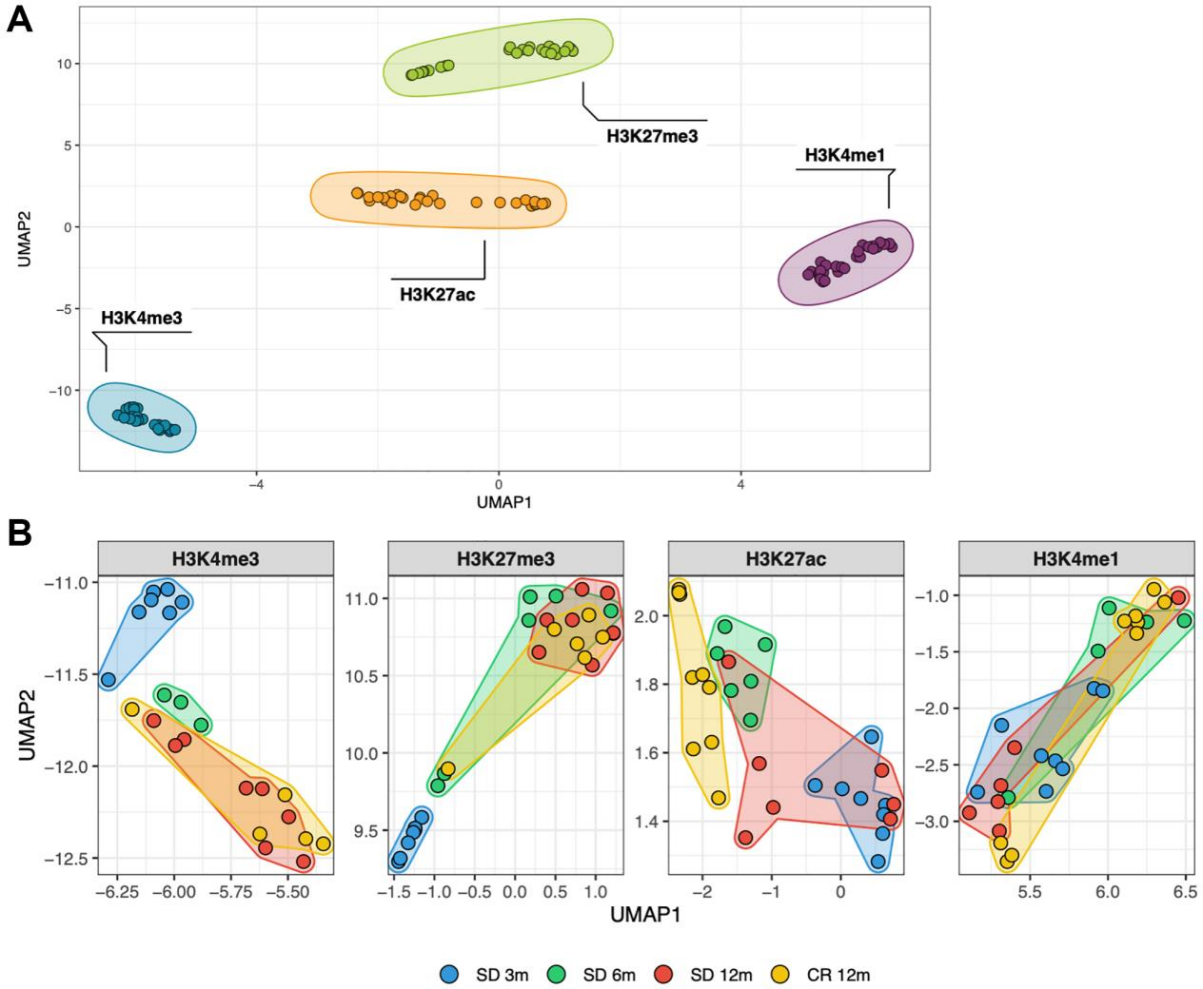
Similarity between all genome-wide histone modifications profiles represented through dimensional reduction using the UMAP algorithm (**A**), samples are colored by histone modification and aggregate primarily by histone mark. (**B**), amplification of previous UMAP with samples split by histone mark and colored by the experimental group they belong to (different age and diet).

With the initial purpose to globally characterize similarities and differences between all the 108 histone modification profiles, considering age and diet as combined factors, a dimensionality reduction technique (Uniform Manifold Approximation and Projection, UMAP) was used to analyze the normalized, genome-wide signal of each respective histone mark in consecutive, non-overlapping bins of 10 kb. The UMAP resulting from that matrix is shown in Figure 1A and 1B. Each dot represents one of the 108 ChIP-seq samples and its relative position in the map considers the signal of all the 10 kb bins of the corresponding animal and histone mark. At a first comparison, including all profiles (Figure 1A), samples clustered largely by histone mark. The heterogeneity among individuals of each group, estimated by the sum of standard deviations in both dimensions, is greater in H3K27me3 (1.65) and H3K27ac (1.37) compared to the other two histone marks, with the H3K4me3 (0.79) profiles being the most homogeneous group (Figure 1A). The comparison between profiles obtained by a single histone mark clearly distinguished younger (blue dots) from older groups (yellow and red dots) in the cases of the H3K4me3 and H3K27me3, but not for H3K4me1 or H3K27ac samples (Figure 1B). Notably, the 6 months old groups (green dots) tend to be located in between the young and old samples, supporting the notion that a temporal trend may exist. From this global analysis, CR profiles (yellow dots) are not distinguishable from the aged-matched SD counterparts (red dots) except for the case of H3K27ac which shows samples separated by diet.

**Figure 2:**
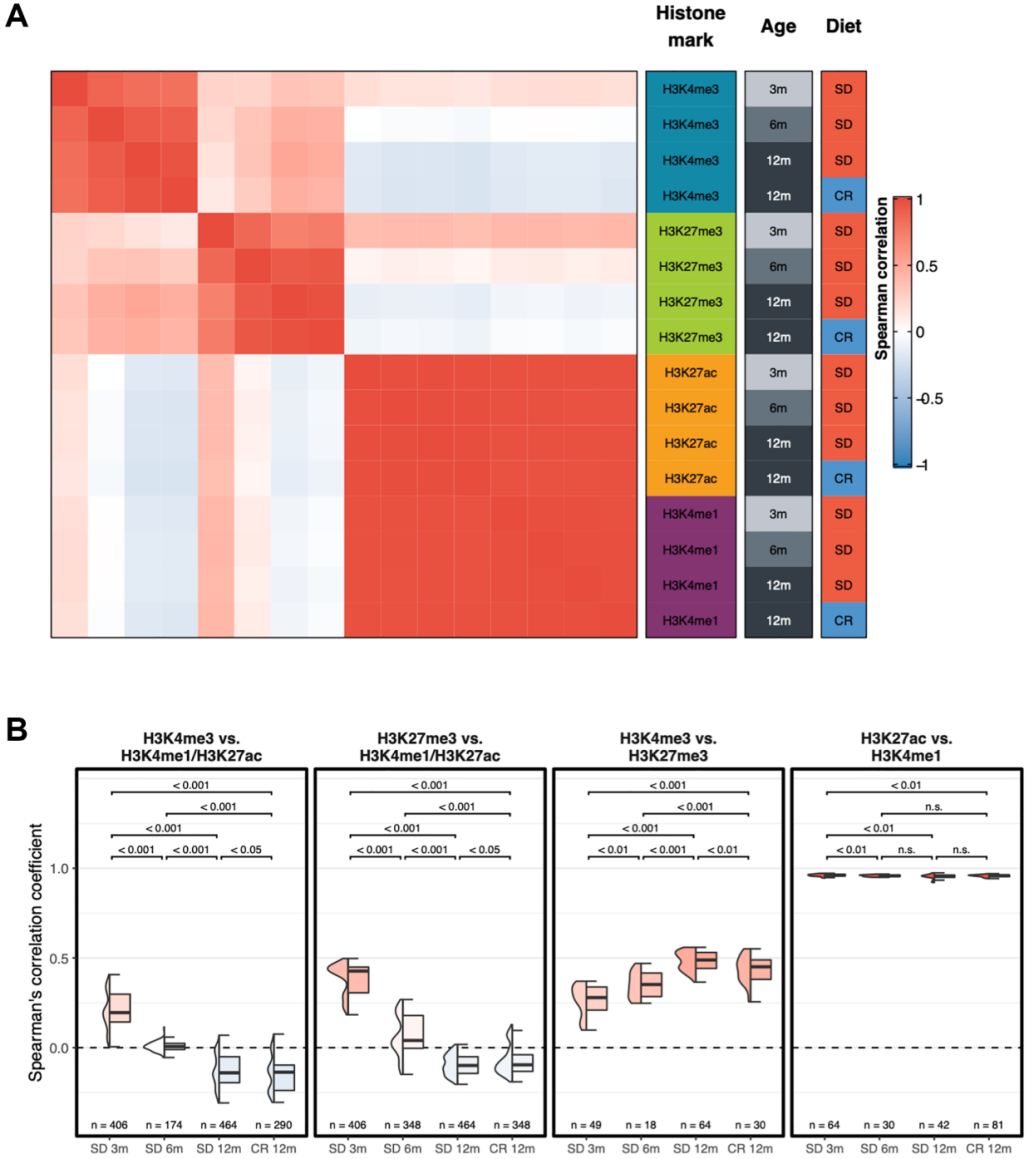
**Genome-wide correlation patterns between histone modifications by group.** (**A**) Heatmap showing the Spearman’s correlation coefficient for each combination of experimental groups after merging the replicates. (**B**) Box plots of Spearman’s correlation coefficients of each replicate between different groups of histone modifications. Two-sided Wilcoxon test *p*-value are shown.

Then, we calculated Spearman’s correlation coefficients between each pair of samples, based on the same genome-wide signal in consecutive, non-overlapping bins of 10 kb, and produced the correlation heatmap between each pair-wise comparison of samples (Figure 2A and Supplementary Figure 1).

Consistent with the proximity seen in first component of the UMAP, the correlation analyses demonstrate that H3K4me1 and H3K27ac groups of profiles were highly similar and are hierarchically clustered into a separate group. Remarkably, unsupervised hierarchical clustering separated the young and old samples (3 months old mice vs. older mice) of both H3K4me3 and H3K27me3 samples. The numbers above the horizontal lines indicate *p*-values from a two-sided Wilcoxon test between the respective groups. Correlation between H3K4me3 (Figure 2B, left panel) and H3K27me3 (Figure 2B, second panel from left) with H3K4me1/H3K27ac profiles decreases with age, unaffected by diet. Correlation between H3K4me3 and H3K27me3 (Figure 2B, third panel from left) increases with age, with a smaller effect in the CR 12m group, while correlation between enhancer-related histone modifications (Figure 2B, right panel) remains high in all groups and does not present an age- or diet-related trend (*p*-value, two-sided Wilcoxon test, are shown above each comparison).

Overall, these findings show that changes occur with time in marking histone H3, in particular with K4me3 and K27me3, in the liver of mice. On a genome-wide scale, promoter-related histone mark profiles and enhancer-related histone mark profiles tend to become more similar with age. Moreover, the promoter-activating and -repressive histone mark profiles become more dissimilar with age, and this phenomenon is partially prevented by caloric restriction (Figure 2B first two panels on the left).

### H3K4me3 and H3K27me3 signal at TSS changes with time

Histone modifications H3K4me3 and H3K27me3 are enriched at the transcription start site (TSS), mostly in a mutually exclusive manner (except for bivalent promoters).

**Figure 3:**
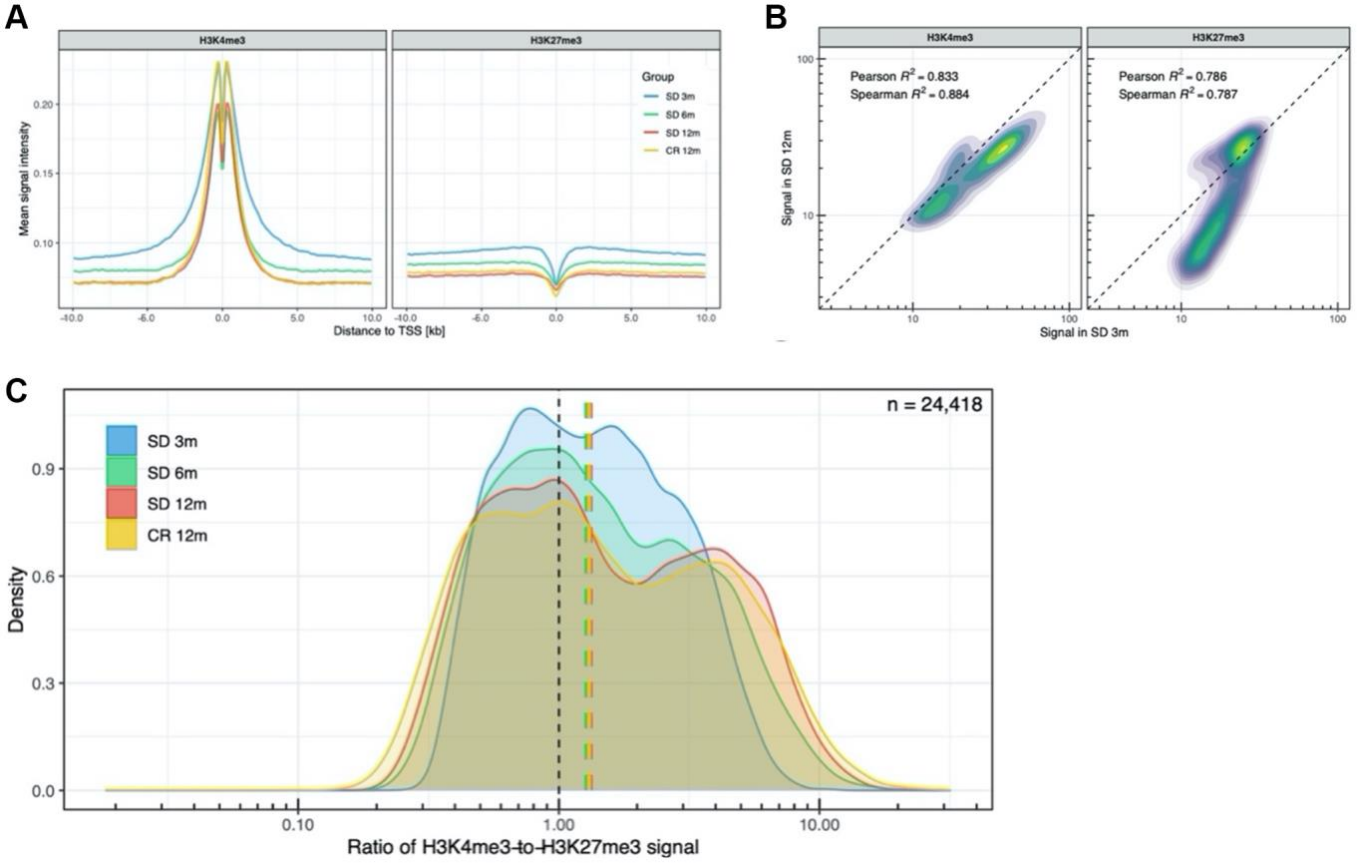
**Analysis of promoter-related histone marks in transcription start sites.** (**A**) H3K4me3 and H3K27me3 profiles around all transcription start sites. (**B**) Density plot of the H3K4me3 and H3K27me3 signal in all TSS, SD 12m over SD 3m. (**C**) Distribution of H3K4me3-to-H3K27me3 signal ratio across all TSS.

To further describe the genomic regions targeted by the time-dependent H3K4me3 and H3K27me3 changes, we focused the analysis on the TSS, comparing the shape of the mean signal of these two histone marks in all 24,418 annotated TSS regions in groups of mice of different age. This analysis revealed consistently stronger (higher amplitude) and wider peaks of H3K4me3 and H3K27me3 in the TSS regions of young mice compared to the older ones (Figure 3A).

Comparing the distribution of H3K4me3 signal between mice aged 3 and 12 months, a slightly decreased signal was observed across most TSS (Figure 3B, left panel). In contrast, the H3K27me3 signal distribution (Figure 3B, right panel) differed more among the two age groups with a subset of TSS having less H3K27me3 in old compared to young mice. We also investigated bivalency of H3K4me3 and H3K27me3 across all annotated TSS regions in young and older samples. As shown in Figure 3C, the ratio of H3K4me3-to-H3K27me3 signal revealed an almost unimodal distribution in 3 months old animals which, with age, assumes a more bimodal distribution in 12 months old mice and an intermediate shape in the 6 months old mice. This transition indicates that the proportion of putative bivalent promoters, having a flexible state, based on the presence of both H3K4me3 and H3K27me3 signal, decreases with time.

### Both age and diet affect chromatin state transitions

**Figure 4:**
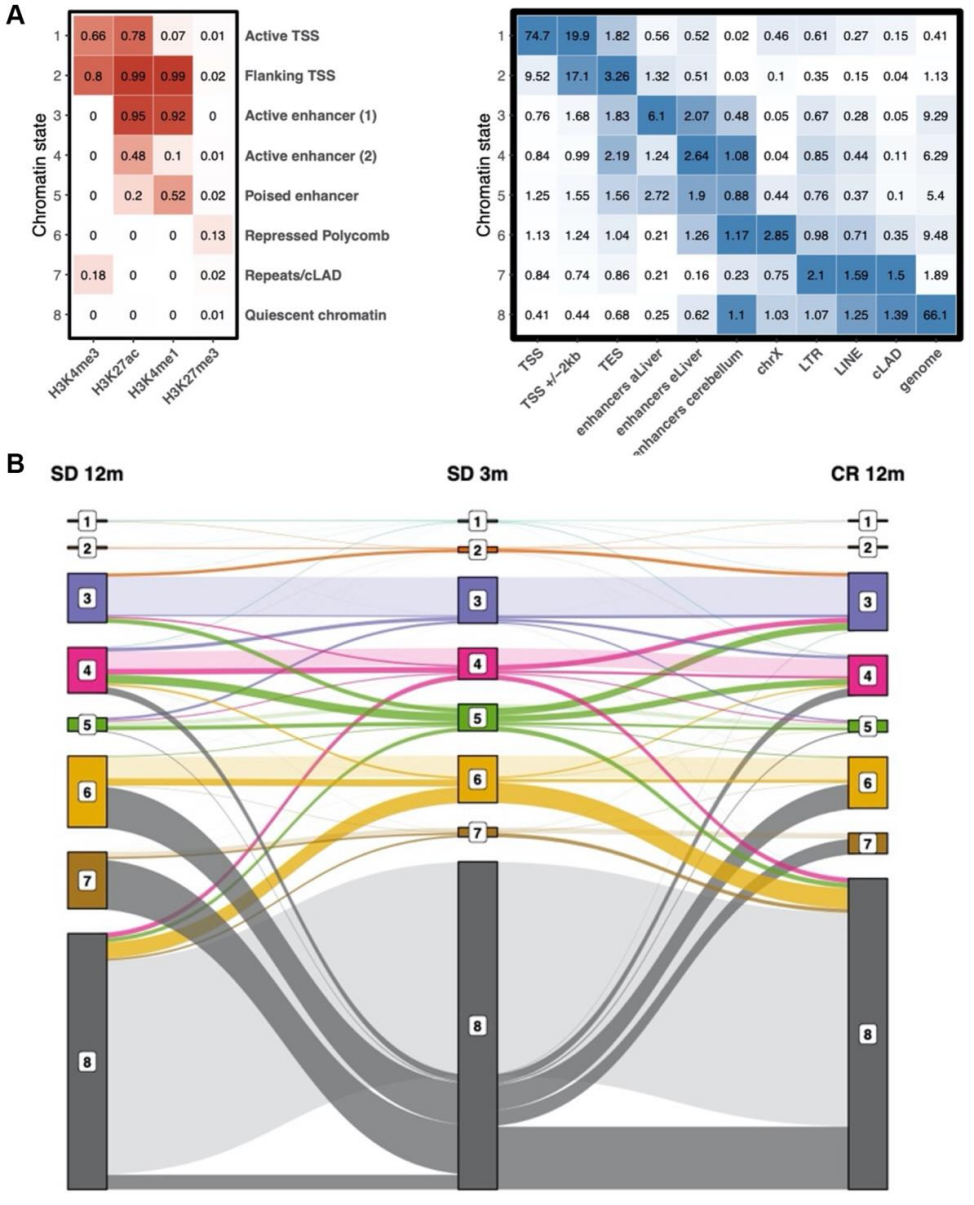
**Chromatin state definition and transitions between states with age.** (**A**) Chromatin state definition, labels and enrichment of selected groups of functional elements in young samples. Number in the heatmap on the left are probabilities of observing a given histone modification in the respective chromatin state. Number in the heatmap on the right are enrichment values obtained by ChromHMM for each combination of chromatin state and feature. (**B**) alluvial plot showing the transition between chromatin states in the SD 3m (center), SD 12m (left), and CR 12m (right) groups. Bar height represents genome coverage in percent (all states together are 100%).

To determine the functional elements which are most affected by the observed time-dependent changes in histone modifications, we performed an integrated chromatin state analysis using all histone mark profiles, taking advantage of the multivariate model approach offered by the ChromHMM tool [27]. In this analysis, chromatin states are defined as recurring combinations of the four studied histone marks in the collected ChIP-seq profiles. Each region of the genome in each of the experimental groups (in consecutive bins of 200 bp) is then assigned one of the chromatin states. As expected, given the histone modifications we investigated, multiple TSS- (state 1 and 2) and enhancer-related (states 3–5) states were readily identified (Figure 4A, left panel). These states are characterized by different combinations of H3K4me3, H3K27ac, H3K4me1, and the absence of H3K27me3. Instead, chromatin state 6 is represented only by the repressive histone modification H3K27me3. State 8 contained none of the histone marks analyzed in this study, and, peculiarly, state 7 presents a new signature of weak H3K4me3 and minimal H3K27me3 signal in the absence of the other two histone marks. Annotation of the identified chromatin states was done based on the combinations of histone marks using related ENCODE studies as a reference [27]. With the exception of chromatin state 7, all other states had been previously detected and annotated in the ENCODE studies.

We validated the learned model by checking which known genomic regions and functional elements are enriched in each chromatin state (Figure 4A, right panel and Supplementary Figure 2). For example, chromatin state 1 presents the classic signature of active TSS and is coherently enriched for TSS regions (RefSeq) as well as CPG islands. Chromatin state 2, previously identified in the ENCODE project as “flanking TSS regions” is enriched in TSS regions +/− 2 kb, transcription end sites (TES), and RefSeq gene bodies. As expected, the known histone modification signature of enhancers (state 3) is enriched in enhancers of the adult liver [28]. Consistently, enhancers identified as active only in the embryonic liver [28] show a stronger enrichment for weak and poised enhancer signatures (states 4 and 5). Apart from embryonic enhancers, also simple repeats show an enrichment in state 5. Chromatin state 6, which is characterized by a distinct H3K27me3 signal, is coherently enriched for Polycomb-repressed chromatin and is the most enriched state on chromosome X. As TSS regions are not enriched in chromatin state 7, even though it contains faint signals of both H3K4me3 and H3K27me3, it is likely not the signature of bivalent promoters. Instead, it is enriched in long terminal repeat retrotransposons (LTR), long interspersed nuclear elements (LINE), and constitutively lamina-associated domains (cLAD) [29]. These functional regions also show enrichment in chromatin state 8 which is characterized by the absence of all four histone marks and covers most of the genome (roughly 50–70%). This finding is consistent with the previously mentioned model from the ENCODE project where the empty chromatin state represents the largest portion of the genome. Notably, the genome column in Figure 4A (right heatmap) represents the genome coverage of each state. As expected, TSS-associated chromatin states 1 and 2 are found only in a very small fraction of the genome. Instead, about 20% of the genome is covered by enhancer-related chromatin states 3, 4 and 5. While state 6 accounts for roughly 10% of the genome, the large majority (more than 60%) is in state 8.

Then, we studied the dynamics by which chromatin states transformed into each other at different age or diverse diet regimen. An alluvial plot helped us to follow these transitions between chromatin states in the SD 3m, SD 12m, and CR 12m groups (Figure 4B). The bar height represents genome coverage in percent (all states together are 100%). Many, but not all, chromatin state transitions observed with age, especially the loss of quiescent chromatin (state 8), are not present or present at a smaller degree in the comparison with the CR 12m group. State 7 shows the biggest fold change between young (SD 3m) and old (SD 12m) mice kept in standard diet. Therefore, we labeled it “age-related state”. Mice kept in caloric restriction show only a slightly elevated genome coverage of state 7 compared to young mice. A similar trend can be observed for state 6, however with smaller changes between young and old SD mice and instead a bigger change between young and old CR samples.

In Supplementary Table 2 we report the percentage of the genome of older groups (6 months and 12 months in both SD and CR) which was found to change chromatin state compared to the youngest group (SD 3m). In all groups, most of the genome (around 68–79%) does not undergo state changes. However, in standard diet, we observed a progressive increase of the portion of the genome which has changed chromatin state, from about 20% in the first three months to more than 30% at 12 months. Remarkably, mice kept in caloric restriction do not show the same extent of state transition (24.2%). Furthermore, we found that genomic regions which were annotated as quiescent chromatin (state 8) in 3 months old mice are assigned either state 4 (active enhancer 2), 6 (repressed Polycomb) or 7 (Repeats/cLAD) in 12 months aged mice depending on the diet (Figure 4B). This means that going from the absence of all four histone marks, the old samples show slight presence of either H3K27me3, H3K4me3 or H3K27ac in the respective genomic regions.

To validate the potential role in aging of the genes whose histones H3 appeared targeted by the observed time- or diet-dependent changes, we used a peak calling tool and differential binding analysis. Ingenuity Pathways Analysis of the differentially H3K4me4-marked genes revealed several pathways involving these gene sets (Supplementary Figure 3). However, no clear gene signatures typical of aging pathways emerged from these gene sets. On the opposite, for the H3K27me3, much less loci showed ages or diet effects (Supplementary Figure 4). We have also investigated presumed active (positive for H3K27ac) or primed (barely positive for H3K27ac and strong for H3K4me1) enhancer regions and enhancer regions identified as previously characterized in the mouse [28] without revealing any different temporal trend or CR effect.

## DISCUSSION

Here, we have characterized the temporal dynamic patterns of four key histone H3 modifications in the liver of healthy C57Bl/6 mice within a year of age to determine whether and to what extend time impacts on chromatin state. Despite lacking mechanistic insights, the collected data indicate a clear drift of histone H3 modifications arising with age in the mouse liver.

Changes in covalent modifications of the histones tails as well as in histone variant incorporation and in DNA methylation have been extensively studied during the development and aging of different organisms [30]. Based on these studies, an extensive chromatin remodelling, which enables the epigenetic reprogramming of tissue functions has been supposed to occur throughout lifespan [224]. However, pure time-dependent (i.e. devoid of the interference of developmental process or aging-associated compliances but reproductive aging) changes in histone modifications have not been systematically assessed in mammals.

In the C57Bl/6 strain of laboratory mice, the time period that follows maturity, starting at 2–3 months of age, is characterized by approximately 6–8 months (depending on the individual) of healthy conditions showing no major signs of frailty. The comparisons we showed (Figures 1 and 2) of the different histone H3 marks profiles obtained at 3, 6 and 12 months of age indicated few months of life were sufficient, from 3 to 6 and from 6 to 12 of age, to identify global changes of K4 and K27 histone H3 trimethylation. Thus, we conclude that extensive temporal changes of H3K4me3 and H3K27me3 occur in a manner that allows the distinction of young and old mice [31]. However, these genome wide changes were not prevented by CR (Figure 2A), which instead, was observed (Figure 2B) to rescue the average signal of H3K4me3 around transcription start sites. Consistently with what has been reported in mammals on the effect of CR on protein [32] and on histone acetylation [33, 34], our analysis revealed that CR particularly affected the acetylation of H3K27 (Figure 1B).

The correlation of H3K4me3 and H3K27me3 to H3K4me1/H3K27ac profiles decreased with age whereas the correlation between H3K4me3 and H3K27me3 profiles increased with age (Figure 2B). Although, the average signal at the TSS regions of both H3 K4 and K27 trimethylation showed an almost linear temporal decay (Figure 3A) and the width of the H3K4me3 peak at TSS collapsed from 3 to 6 months of age. Moreover, at the TSS, the H3K27me3 distribution changed from unimodal at 3 months to a bimodal pattern at 12 months (Figure 3B) suggesting an ongoing process of activating/deactivating genes beyond the developmental period [35].

The mechanisms underlying the vast changes of H3 modifications we observed are not clear. On the biological relevance of these changes, regardless their utility as marker of epigenetic aging, some of the changes observed overtime are prevented by CR suggesting that they may impact on aging phenotypes. The width of H3K4me3 peaks around the TSS region has been linked to transcriptional consistency [36], while changes in H3 K4 and K27 tri-methylation bivalency may affect the stability of the promoter landscape.

The annotation of genomic regions to different chromatin states and investigation of transition patterns between chromatin states among the different age and diet groups revealed that a consistent fraction (20–30%) of the genome changed chromatin state over time. At domain levels, no major differences characterized the groups except for a decreased fraction of poised enhancers. On the contrary, states assigned to quiescent chromatin underwent a massive transition in aged mice which was not observed in mice following the CR diet. The unmarked regions in 3 months old mice were actually lost in older mice kept in SD diet, revealing, together with increased poised enhancer domain, a trend of increased repression and H3K4 tri-methylation on repetitive regions.

Potential biological consequences of altering the control of regions enriched for LTR, LINE and cLAD include activation of mobile elements, altered spatial organization of the chromatin, increased levels of DNA damage and consequent pro-senescence response.

In conclusion, while our data do not establish that the observed changes in H3 modification are causally involved in aging, they indicate age, buffered by caloric restriction, releases the histone H3 marking process of transcriptional suppression in gene desert regions of mouse liver genome most of which remain to be functionally understood.

Such time-related epigenetic changes of chromatin appear more largely due to errors of the epigenetic machinery rather than to functional adaptive process to aged cellular or organismal environment. The underlying idea is that the discovery of these remodeling events and the characterization of the mechanisms which govern them (in the absence of physio-pathological consequences of aging) will represent prototypical models which will help explain the changes that lead to the known phenotype associated with aging.

We are aware of important limitations of the present study: i) sample size may be not sufficient to identify low penetrance phenomena; ii) cell loss and infiltration that are expected in aged tissue may determine variations erroneously attributed to the same cell type.

## METHODS

### Mice

All aspects of animal care and experimentation were performed in accordance with the Guide for the Care and Use of Laboratory Animals published by the US National Institutes of Health (NIH Publication No. 85-23, revised 1996) and the Italian Laws (D.L.vo 116/92and following additions), which enforces EU86/609 Directive. Experiments were approved by the local Ethical Committee, Organismo Preposto al Benessere degli Animali (OPBA), and notified to the Ministry of Health.

Chromatin was extracted from livers collected from inbred C57BL/6 female mice of 3, 6 and 12 months of age fed standard diet (SD) and 12 months of age fed 30% caloric restriction (CR) (Supplementary Table 1). At 3 months of age, the mice were randomly divided in the CR and SD groups and started the CR treatment. To calculate the amount of food in order to provide 30% less calories to the mice with respect to the *ad libitum* SD, food intake was measured daily in a subset of mice from 3 to 6 months of age [37].

All 3 months old females were fertile and plateaued at above 20 grams of body weight. Survival of these females was consistently 100% in the first year. The rare occurrence of injuries, deviant behavior and any external or internal, as determined upon necroscopy, signs of abnormalities including minimal alopecia, dermatitis or histiocytosis, determined the exclusion of the individual from the study. Histological examination of the livers used to extract chromatin did not reveal structural differences in infiltrating cells or residue of hematopoiesis. Altogether, 40 mice were bred and 31 livers collected from 3 months (*n* = 8), 6 months (*n* = 6), 12 months old mice fed SD (*n* = 8) or CR (*n* = 9), and fixed and embedded in paraffin blocks.

### Chromatin immunoprecipitation and sequencing

H3K4me3, H3K27me3, H3K4me1 and H3K27ac profiles were obtained from the aforementioned samples using a modified version of the protocol for extraction of chromatin from formalin-fixed paraffin-embedded (FFPE) tissue samples [38]. Quality control of all ChIPs were performed before sequencing by real-time QPCR, measuring the enrichment observed of bound with respect to the input DNA, on liver transcriptionally active and inactive promoter regions or enhancer regions. Sequencing was performed on an Illumina HiSeq 2000, 50 bp read length, single-stranded.

### Processing of FASTQ files and data analysis

Reads were aligned to the mm10 reference genome using “bwa aln” (v0.6.2-r126). Unmapped reads (reads with a MAPQ smaller than 1), reads that mapped outside of chr1-19 and chrX, as well as duplicate reads were removed using samtools (v0.1.18). Data, if not specified otherwise, was processed inside a Singularity (v2.6.0) container using R (v3.5.1). For some analysis, the BAM files of the replicates of each group were merged using bamtools (v2.5.1), and subsequently indexed using samtools (v1.7). BigWig files for each sample and group were generated from the respective BAM files using deepTools bamCoverage (v3.1.3) with a bin size of 10 bp, BPM-normalization (chrX was ignored for normalization), and reads extended to 200 bp.

Genome-wide signal for UMAP and correlation: for each sample and group, the genome-wide signal was retrieved for consecutive, non-overlapping bins of 10 kb using deepTools multiBigwigSummary (v3.1.3). Uniform Manifold Approximation and Projection (UMAP) was calculated using the genome-wide signal of each sample in bins of 10 kb using the ”uwot” R package. Spearman’s correlation coefficients between samples and groups were calculated using deepTools plotCorrelation (v3.1.3) based on the genome-wide signal of 10 kb bins.

The heatmaps were generated using the “ComplexHeatmap” (v1.20.0) and “circlize” (v0.4.5) R packages. Statistical significance shown in the box plots were generated using the “ggsignif” (v0.4.0) R package using a Wilcoxon test.

### TSS profiles, signal and ratio distribution and enhancer signal

Annotation of transcription start sites (TSS) was taken from the curated RefSeq set, downloaded from UCSC Genome Browser on 01.03.2018. The list of TSS includes the TSS of the longest transcript for each gene. The signal around TSS was calculated as mean signal in bins of 50 bp, with a range of +/− 5 kb around the TSS, using deepTools “computeMatrix” (v3.1.3). Missing data was treated as zero. The output was then plotted (for profile) or summed up by TSS to generate the overall signal. For the ratio, summed signal values for H3K4me3 signal was divided by the H3K27me3 signal for each individual TSS. Relative levels of H3K4me3 signal on selected TSS regions obtained upon ChIP-seq from comparing 3 months with respect to 12 months old samples were validated by investigating gene expression fold by reverse transcriptase-QPCR on same samples (Supplementary Figure 5).

### Chromatin state analysis

Chromatin state analysis was performed using ChromHMM (v1.17). Annotation of TSS represents the same list of TSS as above with the range adjusted to +/− 2 kb. Similarly, annotation of transcription end sites (TES) was taken from the curated RefSeq set, downloaded from UCSC Genome Browser on 01.03.2018. The list of TES includes the TES of the longest transcript for each gene. Annotation of chrX was taken from the mm10.txt file provided with ChromHMM. Long terminal repeats and long interspersed nuclear elements annotation was downloaded from the UCSC Genome Browser for mm9 and then converted to mm10 using the UCSC Genome Browser LiftOver feature. Annotation of constitutively lamina-associated domains (cLAD) was taken from the report of Peric-Hupkes et al. [39] and converted from mm9 to mm10 using the UCSC Genome Browser LiftOver feature (mm9 files can be found in the GSE17051 data set in the GEO database). Merged BAM files of each group were binarized using the “BinarizeBam” command. Then, the chromatin state model was trained using the “LearnModel” command for 8 outcome states. States were then reordered using the “Reorder” command, then segmentation and enrichment with sets of genomic locations were generated using the “MakeSegmentation” and “OverlapEnrichment” commands. For the transition analysis, BED files of all groups containing the chromatin state for every location in the genome were cut into bins of 200 bp using awk (v4.0.2) and bedtools (v2.26.0). Alluvial plot was generated with the “ggalluvial” R package (v0.9.1).

More information about chromatin states can be found at http://compbio.mit.edu/ChromHMM/ and https://www.encodeproject.org/publications/aa02932b-39c5-43d8-9ee1-2059fb604a81/.

### Data availability

ChIP-seq data are accessible on GEO repository (https://www.ncbi.nlm.nih.gov/geo/) with accession number GSE179090. All the data that support the figures and the other findings are available from the authors upon request.

## Supplementary Materials

Supplementary Figures

Supplementary Tables
