# Supplementary material for: Time makes histone H3 modifications drift in mouse liver
Source: Aging (Albany NY). 2022 Jun 10;14(12):4959–75. doi: 10.18632/aging.204107 (PMC9271290; doi:10.18632/aging.204107)
Supplement: Supplementary Figures [file aging-14-204107-s001.pdf]

## SUPPLEMENTARY FIGURES

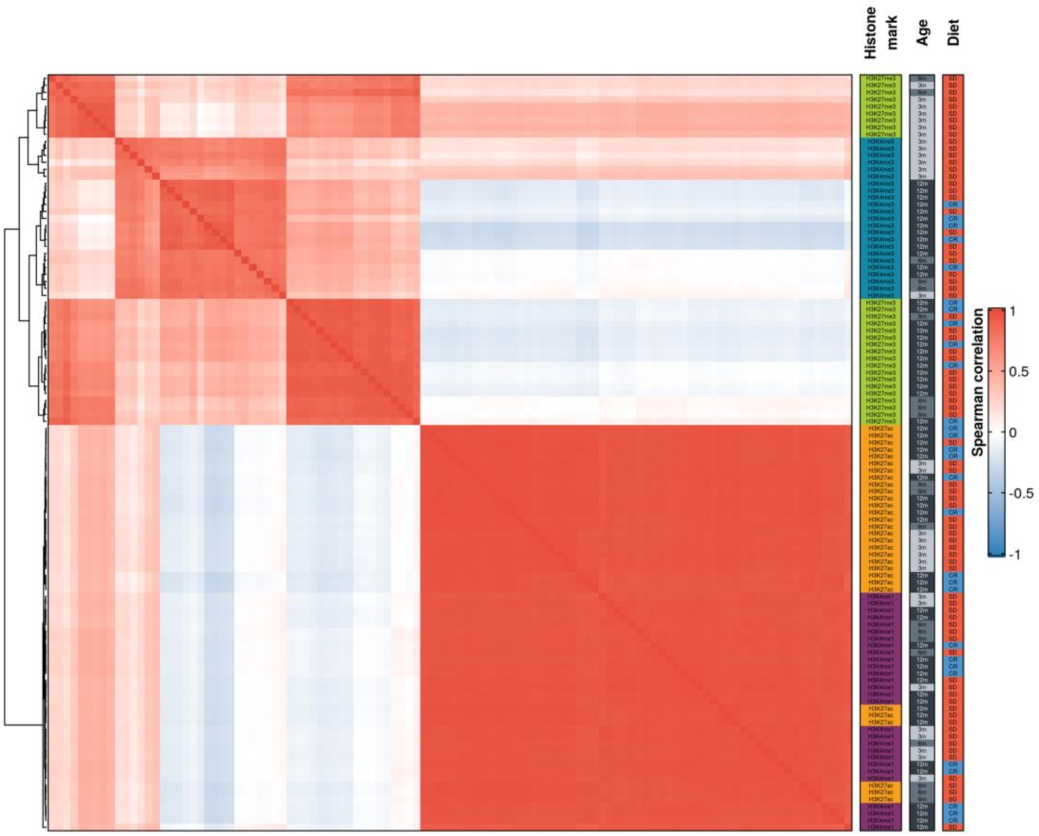


**Supplementary Figure 1. Heatmap of genome-wide correlation patterns between all 108 histone modification profiles.** Genome-wide histone modification profiles of all 108 samples were cut into consecutive, non-overlapping bins of 10 kb to assess correlation between each pair of samples. Heatmap shows the Spearman's correlation coefficient for each pair-wise comparison of individual samples.

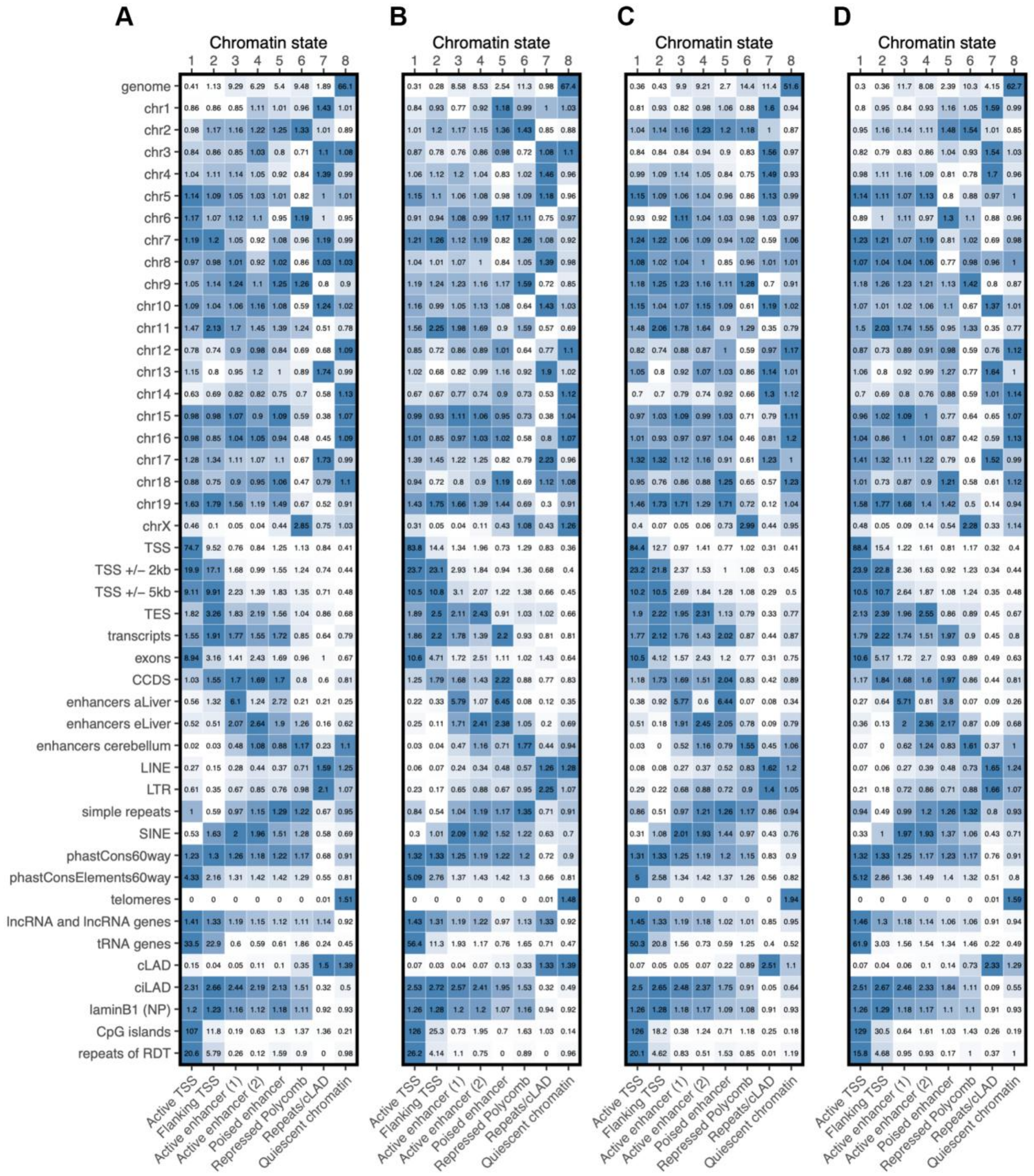


**Supplementary Figure 2. Enrichment of chromatin states in annotated genomic features.** The annotated genomic regions and functional elements enrichment is calculated by ChromHMM algorithm using the equation: enrichment = (C/A)/(B/D); with (**A**) number of bases in the state; (**B**) number of bases in the external annotation; (**C**) number of bases in the state and the external annotation; (**D**) number of bases in the genome. Numbers in the heatmap represent the enrichment values returned by ChromHMM. Instead, the color is independent for every row in every panel, highlighting the minimum and maximum value for each feature. Enrichment was calculated separately for the SD 3m (**A**), SD 6m (**B**), SD 12m (**C**), and CR 12m (**D**) groups.

## H3K4me3

**A**

| Comparison | Total db regions | Fold > \|0.5\| | No Distal |
| --- | --- | --- | --- |
| sd_03_sd_06 | 5297 | 2937 | 1967 |
| sd_03_sd_12 | 12541 | 7547 | 6910 |
| sd_12_cr_12 | 19 | 19 | 18 |

**B**

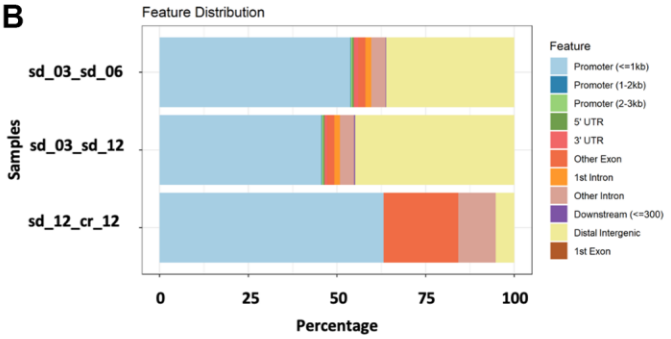


**C**

| geneId | log2(fold) |
| --- | --- |
| Gm129 | 1.205196 |
| Parp4 | 1.204001 |
| Map2k6 | 1.072883 |
| Hdc | 1.025854 |
| Ces1d | 1.009268 |
| Per2 | 0.923239 |
| Dbp | 0.873131 |
| Ccrn4l | 0.844284 |
| Ahcy | 0.819705 |
| Cyp2c54 | 0.80147 |
| A430090L17Rik | 0.731856 |
| Slc38a4 | 0.63839 |
| Dlc1 | -0.7913 |
| Stat5b | -0.95018 |
| Mir1903 | -1.02546 |
| Cyp2c44 | -1.20169 |
| Dio1 | -1.23997 |
| Ano2 | -2.52633 |

**D**

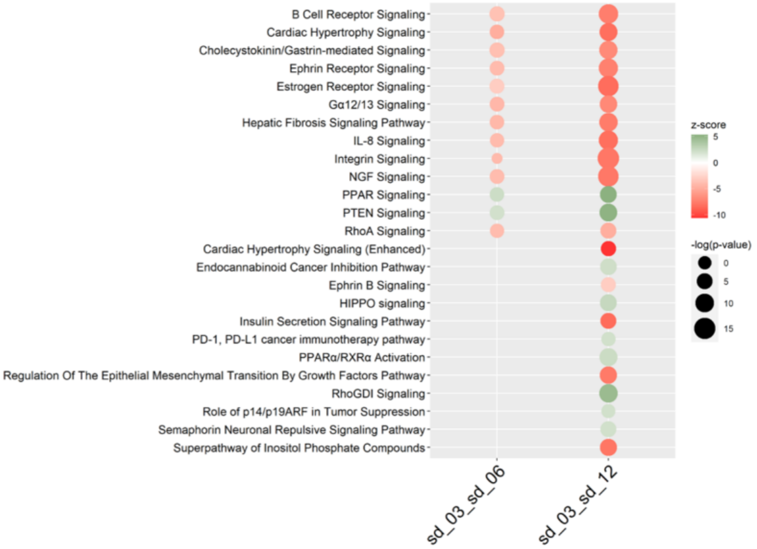


**Supplementary Figure 3. Differential binding analysis.** Peak detection was performed with epic2 software using the following parameters: bin = 200, g = 2, and e = 100. Differential binding analysis was performed by the Diffbind (v3.0) R package, using TMM normalization on background and edgeR for differential analysis. Stringency in the analysis was obtained by creating a consensus dataset for each condition, including peaks that were present in at least 2 samples. Only DB sites with an FDR ≤ 0.05 were considered. The ChIPseeker R package was applied to annotate DB sites using annotation from curated RefSeq set, while pathway analysis was conducted using the Ingenuity Pathway Analysis (IPA) software from QIAGEN. Pathways with an absolute z-score >2 and –log (*p* value) > 1.3 were considered significant. (**A**) Number of H3K4me3 differentially marked sites found for each comparison using the Diffbind R package and differentially bound sites with fdr ≤ 0.05 were reported. (**B**) Annotation of the H3K4me3 DB sites found for each comparison. ChIPseeker R package was applied to annotate H3K4me3 DB sites found in each comparison. (**C**) List of the 18 genes found for the comparison SD12m vs. CR 12m. (**D**) Ingenuity pathway analysis (IPA). IPA analysis was carried out using only differential enriched regions associated to genes. First 10 upregulated terms were selected individually for each comparison and, when significant, reported for both the analyses.

## H3K27me3

**A**

| Comparison | Total db regions | Fold > \|0.5\| | No Distal |
|---|---|---|---|
| sd_03_sd_06 | 41 | 26 | 18 |
| sd_03_sd_12 | 220 | 93 | 53 |
| sd_12_cr_12 | 4 | 1 | 0 |

**B**

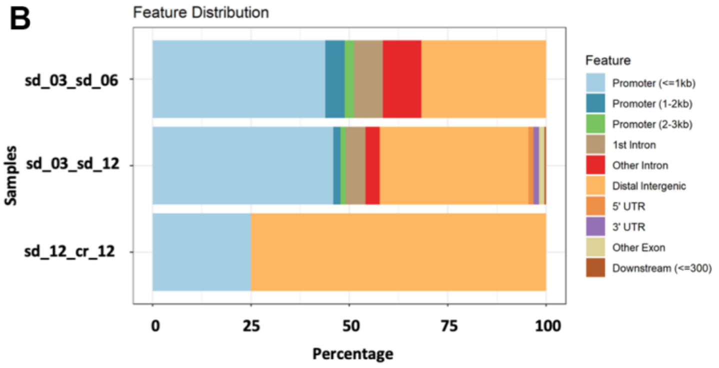


**Supplementary Figure 4. Differential binding analysis. Peak detection was performed with epic2 software using the following parameters: bin = 200, g = 3 and e = 100.** Differential binding analysis was performed by the Diffbind (v3.0) R package, using TMM normalization on background and edgeR for differential analysis. Stringency in the analysis was obtained by creating a consensus dataset for each condition, including peaks that were present in at least 3 samples of each group. Only DB sites with an FDR ≤ 0.05 were considered. The ChIPseeker R package was applied to annotate DB sites using annotation from curated RefSeq set. (**A**) Number of H3K27me3 DB sites found for each comparison. Differential binding analysis was performed using the Diffbind R package and differentially bound sites with fdr ≤ 0.05 were reported. (**B**) Annotation of identified H3K27me3 DB sites found for each comparison. ChIPseeker R package was applied to annotate H3K27me3 DB sites found in each comparison.

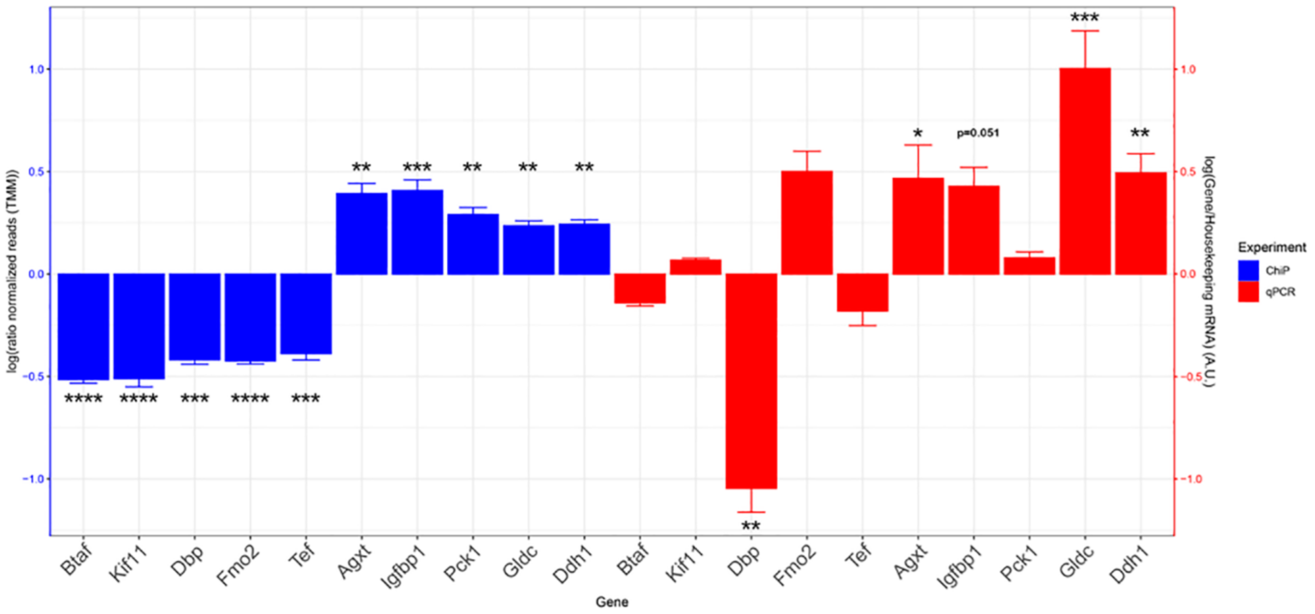


**Supplementary Figure 5. H3K4me3 signal and RNA expression values of selected genes.** The log ratio of normalized counts (TMM) of H3K4me3 signal of 5 top "upregulated" (bars with positive scores) and 5 top "downregulated" (bars with negative scores) promoter sites in 12 months fed SD vs. 3 months old samples is reported in blue (left side of the plot). The log fold change of the RNA expression of the same genes is represented in red (right side of the plot). Bars with positive scores indicate upregulated genes, bars with negative scores indicate downregulated genes in 12 months fed SD vs. 3 months old samples. Significance is calculated with a *t*-test. $^{*}p$ value < 0.05, $^{**}p$ value < 0.01, $^{***}p$ value < 0.001, $^{****}p$ value < 0.0001.
