# Supplementary material for: Time makes histone H3 modifications drift in mouse liver
Source: Aging (Albany NY). 2022 Jun 10;14(12):4959–75. doi: 10.18632/aging.204107 (PMC9271290; doi:10.18632/aging.204107)
Supplement: Supplementary Tables [file aging-14-204107-s002.pdf]

SUPPLEMENTARY TABLES

Supplementary Table 1. Number of available samples across all groups of this experiment.

	SD 3m	SD 6m	SD 12m	CR 12m	Total
**H3K4me3**	7	3	8	5	23
**H3K27me3**	7	6	8	6	27
**H3K27ac**	8	6	7	9	30
**H3K4me1**	8	5	6	9	28
**Total**	30	20	29	29	108

Supplementary Table 2. Fraction of the genome that changes chromatin state compared to the young (SD 3m) group.

	Fraction of genome with same chromatin state	Fraction of genome with different chromatin state
SD 6m	78.9%	21.1%
SD 12m	68.1%	31.9%
CR 12m	75.8%	24.2%

The percentage of the genome of older groups (6 months and 12 months in both SD and CR) which was found to change chromatin state compared to the youngest group (SD 3m).
